# Can the Administrative Loads of Physicians be Alleviated by AI-Facilitated Clinical Documentation?

**DOI:** 10.1007/s11606-024-08870-z

**Published:** 2024-06-27

**Authors:** Henry Bundy, Jay Gerhart, Sally Baek, Crystal Danielle Connor, McKenzie Isreal, Ajay Dharod, Casey Stephens, Tsai-Ling Liu, Timothy Hetherington, Jeffery Cleveland

**Affiliations:** 1 Center for Health Systems Sciences, Atrium Health Wake Forest Baptist, Winston-Salem, NC USA; 2 The Innovation Engine at Atrium Health, Charlotte, NC USA; 3 Department of Internal Medicine, Wake Forest University School of Medicine, Winston-Salem, NC USA; 4 Information Technology, Advocate Health, Oak Brook, IL USA

## Abstract

**Background:**

Champions of AI-facilitated clinical documentation have suggested that the emergent technology may decrease the administrative loads of physicians, thereby reducing cognitive burden and forestalling burnout. Explorations of physicians’ experiences with automated documentation are critical in evaluating these claims.

**Objective:**

To evaluate physicians’ experiences with DAX Copilot (DAXC), a generative AI-facilitated clinical documentation tool.

**Design:**

Semi-structured interviews were conducted in August and September of 2023 with physician-users of DAXC.

**Participants:**

A purposive sample of 12 interviewees, selected from 116 primary care physicians, employed at a multi-site academic learning health system.

**Approach:**

After completing all 12 interviews, three study personnel independently analyzed and coded the transcripts. Reconciliation sessions were then held to merge the three analyses into one summary, eliminating redundant codes, and grouping findings into themes.

**Key Results:**

For a majority of interviewees, DAXC reduced the amount of time spent documenting encounters, and alleviated anxieties of having to retain important clinical details until there was time to make notes. DAXC also allowed physicians to be more engaged during appointments, resulting in more personable provider-patient encounters. However, some physicians weighed these benefits against an uneasy feeling that interviewees might be asked to see more patients if DAXC was mandated. Physicians also noted that the tool would occasionally imagine or misgender patients, offer unsolicited and inappropriate diagnoses, and mistake critical details in transcription. The few physicians less enthusiastic about the generative technology portrayed themselves as creatures of habit who had cultivated long-standing workflows and particular notation practices that DAXC could neither improve upon nor reproduce.

**Conclusions:**

According to physician interviewees, automated AI-driven clinical documentation has the potential to significantly reduce the administrative burden associated with particular types of provider-patient encounters. Addressing the growing pains of the incipient technology, identified here, may allow for a broader applicability for clinical practice.

**Supplementary Information:**

The online version contains supplementary material available at 10.1007/s11606-024-08870-z.

## INTRODUCTION

The emergence of AI-facilitated primary care documentation has long been anticipated.^1–3^ With the advent of new powerful large language models, the potential for artificial intelligence to automatically draft clinical notes has increased considerably.^4^ Yet, it is unclear whether AI-facilitated clinical voice technology can reduce the cognitive burdens and documentation loads of physicians, as promised.^5^

The following is a qualitative exploration of primary care providers’ experiences with DAX Copilot (DAXC), a generative clinical voice technology facilitated by artificial intelligence (AI). The emergent health technology is meant to reduce the administrative burden on healthcare providers by employing Generative Pre-trained Transformer 4 or GPT-4 (OpenAI 2024) to document and transcribe patient-provider interactions in real time. DAXC uses conversational generative AI to automatically and instantaneously draft clinical notes after a patient visit. This qualitative evaluation was a component of a larger mix-methods evaluation of the DAXC application within Atrium Health. The study is funded by Wake Forest University Health Sciences (ClinicalTrials.gov number, NCT06329427).

DAXC is owned by Nuance, a Microsoft company (Nuance 2024). The tool has evolved and is now integrated into several EHRs. During this pilot, the DAXC provided narrative summaries. It is anticipated that the tool will eventually provide additional automation within the EHR by initiating other workflows based on the conversation between a patient and clinician.

On the commercial website of DAXC, champions of AI-based clinical documentation assert the application’s potential to reduce physician burnout, streamline workflows, and increase the accuracy of provider notes.^6^ Advocate Health (AH), a multi-site academic learning health system, is an early adopter of DAXC. The objectives of the following AH-based qualitative evaluation were (1) to gather providers’ perspectives on, and experiences with, DAXC; (2) to assess the impact of the generative clinical voice technology on the workflows and workloads of AH providers; and (3) to determine how the technology could most effectively be incorporated into practice at AH.

The typical DAXC workflow involves several steps. The clinician will usually open the DAXC application and start a recording prior to entering a patient’s room. Information about the visit (the reason for visit, the patient’s medical history, etc.) may be recorded before pausing the recording. Upon entering the patient’s room, the clinician will request verbal consent from the patient to have DAXC listen in on the visit. If consent is not permitted, the clinician proceeds with the patient visit without the aid of DAXC. If consent is received, the clinician resumes their initial recording and proceeds with the patient visit. As the patient visit concludes, the clinician will stop the recording, and a preliminary clinical note is drafted within 30 s for review. The clinician can then edit the drafted note and finalize it within the electronic health record (EHR) or on their mobile phone.

## METHODS

Between August 17 and September 13, 2023, we conducted semi-structured interviews with 12 primary care physicians. Interviewees were selected from among the 116 AH physicians recruited to pilot DAXC. The literature on administrative burden among physicians suggests that they are at higher risk of administrative burden-related burnout if they are early-career physicians (≤ 10 years since training), women, or minority providers.^7,8^ Consequently, interviewees were selected using a purposive sampling strategy, which sought diverse physician experiences.

During the data collection phase of the project, the study team met regularly to parse emergent results and discuss potential interview themes. According to notes taken during these discussions, no new themes emerged after the 10th interview, and by the 12th, interviewers noted that responses to questions had become consistently redundant, and that thematic saturation had appeared to have been reached.

This sample size (*n* = 12) corresponded with our rough estimate of the potential information power inherent in our interviewee group.^9^ Our estimation considered the amount of pertinent information available, the depth and breadth of inquiry possible, the probable diversity of experiences present, the size of the prospective interviewee pool, and the breadth of the study aim. Ultimately, 12 interviews proved sufficient, a consequence of the project’s narrow study aim, and its high-information interviewee population.

Interviews were conducted remotely, via Microsoft Teams, and lasted, on average, around 30 min. None of the primary care providers selected for interviews declined to be interviewed. Interviews were transcribed by H.B., a senior health services researcher at AH. The study team used the qualitative software ATLAS.ti v.9 (ATLAS.ti Scientific Software Development GmbH) to organize, parse, and collate interview data and Miro, a collaborative whiteboard application (Miro Enterprise) to map and organize codes, quotes, and themes.

Our analysis was inductive and iterative, an approach well-suited for exploratory examinations of novel or under-studied social phenomena. Interview data was coded both “horizontally,” by comparing and contrasting specific lines of inquiry across interviews, and “vertically,” by examining individual interviews in their entirety.^10^ This methodical parsing of the data was done repeatedly, as early, contingent codes, were regularly renamed, merged, divided, or deleted all together. Once the code list was solidified, themes, summative propositions used to make sense of and connect recurring ideas in a study, were induced through the repeated parsing and partitioning of the interview data. J.G. and S.B., members of the AH Innovation Engine, developed the initial codes and oversaw the visualization and mapping of the interview data. H.B., D.C., and M.I., members of AH’s Center for Health Systems Sciences, produced the final themes of the analysis.

Once completed, the interviews were analyzed by two members of the study team. The analysts independently reviewed the recordings and coded the transcripts. After completing all 12 interviews, a three-person team held reconciliation sessions to merge the two analyses into one summary, eliminating redundant codes, and grouping findings into themes. A separate team, made up of three study team members not involved with the initial interviewing and coding, reviewed the resultant themes and evaluated them against the recordings and transcripts.

The semi-structured interviews were part of a larger evaluation for our enterprise. The results of the interviews contributed to the overall evaluation of DAXC and informed the decision of expanding utilization of the tool across the enterprise. This study was approved by the Atrium Health Wake Forest Baptist IRB.

## RESULTS

The principal results of our qualitative analysis fall into three broad themes—the potential or realized benefits of DAXC, the encounters for which the AI technology is suitable, and physician’s concerns with AI-facilitated clinical documentation (see Table 1). The majority of physicians we interviewed reported that DAXC reduces the amount of time spent on clinical documentation, relieving cognitive burden and facilitating more engaged care visits. However, interviewees noted that the AI technology may not be appropriate for documenting all types of encounters, and in some cases DAXC documentation falls short of physicians’ standards for notation (Table 2).

**Table 1:** Interview Themes

Theme 1: Benefits of DAXC	
Improved physician’s quality of life (*n* = 10)	“Weeknight and Saturday charting used to take 2.5–3 h and DAX cut it to 1 h… I sleep more and value my Saturday mornings with my kids.”
Significantly reduced the time physicians spent working after regular hours (*n* = 7)	“Out of the week now, it may be one day that I have to come home and finish notes, but it's not taking three and a half hours…at most an hour and a half.”
DAXC relieved some of the cognitive burden of clinical work (*n* = 6)	“I’ve noticed that my emotional tank is much fuller at the end of the day with DAX than it was before.”
DAXC allows physicians to be more attentive and personable during patient encounters (*n* = 4)	“If I am with someone and DAX is running, I’m sort of knee to knee with them, you know, physically, proximately closer and then definitely much more present…it lets me really listen and take in what [patients] are talking about.”
Theme 2: Suitable encounters	
Some physicians find DAXC best suited for more complex patient encounters (*n* = 6)	“I didn’t want to use [DAXC] for very simple sick visits, because in those visits I can get in and out of the room in less than 10 min with documentation complete.”
Others believe that DAXC is best suited for specific and bounded issues (*n* = 3)	“[DAXC is] especially useful when patients have an urgent care problem, and it’s very specific.”
Theme 3: Concerns about DAXC	
DAXC make errors and can mistake the context of patient encounters (*n* = 6)	“Sometimes the patient will say ‘I did not’ and [DAXC] would say the patient ‘did.’ So that’s a huge issue and I kept seeing that a lot.”
The notes DAXC produces can, at times, be overwhelming (*n* = 5)	“DAX tends to include superfluous details which may dilute the reasons for visit.”
DAXC lacks sensitivity (*n* = 3)	“I feel like [DAXC] misses some of the physical exam things, unless I explicitly say I’m going to look in your ears, I kind of wish it was able to recognize the more subtle hints and clues I’m giving.”
Physicians worried that the implementation of DAXC would result in having to see more patients (*n* = 3)	“As long as they don’t try to make me see more patients, I’m good.”

**Table 2:** DAXC User/Interviewee Profile

No	Specialty	Gender	Type notes in room?	Pre-DAX note process	Current DAX usage
1	Internal medicine	F	Yes	Start template 30–60 s prior to visit, some notes in room and immediately after; finish notes at home	Uses 100% of time
2	Internal medicine	M	Some	Pre-charts, brief notes during visit, some dictation after; significant time in evenings and weekend	Unclear, seems to use for most if not all visits
3	Internal medicine	F	No	Does pre-charting at 5:30; completes most notes right after visit; more complex later in the day	Uses for most visits, except wellness visits and simple visits such as wound checks
4	Family medicine	M	No	Dictate portion of notes immediately after visit, finish at home in evening	Using for non-routine visits; does not use assessment and plan sections
5	Family medicine	M	Yes	Completes most of notes within room, finishes in 30–60 s after leaving	Uses for only ~ 10% of visits, more complex or patients that are not theirs; DAX was less efficient for the vast majority of patients
6	Family medicine	M	Yes	Completes potion of notes within room; remainder throughout day, evening, or next morning	Uses for 95% of visits
7	Family medicine	F	Yes	“Big pre-charter”; bullet point HPI in room, takes 2–3 min to finish assessment and plan before next pt	None, stopped using after 4 weeks
8	Family medicine	F	No	Document notes after visit, batching visits 30–120 min later, finish after work or at home in evening	Unclear, seems to use for most if not all visits
9	Family medicine	M	Yes	Typed in some notes during visit, immediate priorities after, finish note over the weekend	Use 100% of the time
10	Pediatrics	M	Yes	Types most of note within room; completes most right after visit; remainder at lunch, 30–60 min EOD	None, stopped using after 2 weeks
11	Pediatrics	M	Yes	Typed most notes during the visit, some later in the day or evening	Does not use often for well visits, but glad when they do
12	Pediatrics	M	Only some written notes	Write notes in room on pt. information form, then dictate notes into chart outside room; does not pre-chart	Uses for sick visits, not for well visits b/c Dragon smart texts are so efficient (uses for 1/3 to 1/2 of visits)

### The Benefits of DAXC

Physicians experienced or foresaw three benefits of implementing DAXC, an improvement of their quality of life, a reduction of their daily cognitive burden, and an improvement in the quality of their engagement with patients.

Most interviewees (*n* = 10) saw, or assumed DAXC would result in, improvements in their quality of life. “Overall, I’m going to get more sleep, I’m going to feel less stressed,” Provider D, who had been using DAXC for nearly 7 weeks, speculated. For physicians who still dictated notes at home, like Physician J, [DAXC] significantly reduced the time spent working after regular hours. “Out of the week now, it may be one day that I have to come home and finish notes, but it's not taking three and a half hours…at most an hour and a half.” These interviewees noted that by automating notation, DAXC relieved some of the cognitive burden of clinical work. “[At Clinic X], we’re all constantly on the edge of some level of burnout. And I think this hasn't ended because [of DAXC], but what it does do is make me feel a little bit less burned out,” Physician E noted.

Several interviewees (*n* = 5) also said that because they were often backlogged and overwhelmed, they regularly experienced a fear that they might forget an important clinical detail before they could get to their notes. “You really get this sort of burned-out feeling, kind of, helpless,” Physician H explained, “I got to get through this and if I don’t get through this, it’s going to stack up more the next day and I'll be remembering even less than I am right now.” This feeling, interviewees noted, abated substantially once they began using DAXC. “[DAX] helps a lot with cognitive offloading,” Physician F noted, “So it’s not as big of a difference if I’m [writing notes] right after the visit versus doing it a few hours later versus doing it the next day…Most of the information is there…you’re not going to lose information like you might have before.”

DAXC also allowed interviewees to be more attentive and personable during patient encounters. “If I am with someone and [DAXC] is running,” Physician B explained, “I’m really sort of knee to knee with them, physically, proximately closer and definitely much more present.” With DAXC, physicians could also make more eye contact with their patients. “I think the times that I use [DAXC] in the room, I'm having the opportunity to look directly at them without having the computer between us,” Physician F said. This arrangement fostered trust and understanding, Physician E explained, “[Making] eye contact the whole time…is essential for building a strong rapport for having a real conversation building trust…I’m hearing what [patients are] saying at a deeper level.”

### Suitable Encounters for DAXC

While physician-interviewees agreed that DAXC was more suitable for some encounters than others, they differed on the kind of encounters they felt were most appropriate for the tool. For some interviewees (*n* = 3), DAXC was more useful when a patient’s complaint was certain and circumscribed. “[DAXC is] especially useful when patients have an urgent care problem, and it’s very specific,” Physician C noted, “It’s more challenging for maybe an 85-year-old female patient, that comes in mainly for a social visit. Those are a little more difficult.” To complicate things further, DAXC also had trouble dictating notes in chronological order, Physician J said, as patients often narrated their health complaints out of sequence, “DAXC will transcribe [the patient’s narrative] in the order they tell it. If I were to dictate, I would do it in chronological order.” Others (*n* = 6), like Physician A, did not find it worthwhile to use DAXC for short visits. “I didn’t want to use [DAXC] for very simple sick visits,” they said, “because in those visits I can get in and out of the room in less than 10 min with documentation complete.”

### Concerns About DAXC

The concerns of physicians fell into three broad sub-categories: DAXC transcripts could be verbose and include consequential errors, and physicians worried that the implementation of the clinical voice technology would result in being asked to increase their patient volume.

Some interviewees (*n* = 4) did not find DAXC an improvement over their own, well-honed processes. At times, for example, the AI would conjure additional patients or contrive imagined events. “[I've caught it] on occasion making stuff up,” Physician G said, “like saying there are several people in the room when there aren’t, or writing down events that never actually happened.” Mistakes like this would require laborious editing from interviewees. The tool would also misgender patients on occasion, “Sometimes there can be confusion about the gender of the patient…if it’s a woman that has a lower tone of voice,” Physician C recalled. Furthermore, DAXC regularly mistook important clinical details, Physician L noted, “Sometimes the patient will say ‘I did not’ and [DAXC] would say the patient ‘did.’ So that’s a huge issue and I kept seeing that a lot.” DAXC would also occasionally draw unsolicited and inappropriate conclusions. “It throws in random diagnosis that are not even remotely related to something that we were discussing,” Physician D said.

Many physicians (*n* = 7) also noted that the notes DAXC produced could, at times, be overwhelming. “[DAXC] can be rather verbose,” Physician K noted, “…this person has a common cold and it generated six paragraphs.” This verbosity, interviewees believed, was the result of DAXC’s inability to, at times, distinguish pertinent information from irrelevant exchanges. “Yesterday,” Physician A said, by way of example, “a guy brought his wife in to get stitches out, but he went and talked about his own issues…that really confused [DAXC]. So that was a lot of chopping out of unnecessary dictation.” Finally, some physicians (*n* = 3) worried that the implementation of DAXC would result in having to see more patients. “I do worry that’s what this long game is, if you can make doctors more efficient, then you’re going to drop more on us. I do not support that. That makes me nervous,” Physician H, said.

## DISCUSSION

Several themes emerged from our qualitative analysis. Interviewees concurred that DAXC had the potential to reduce time spent on clinical documentation, which they said could, in turn, relieve some amount of cognitive burden and allow for more personable and attentive patient care. Physician-interviewees also agreed that DAXC was more suitable for some encounters than others, but often differed on the kind of patient visit they believed most appropriate for the tool. Some physicians found the technology to be more effective for short, simple encounters, while others preferred it for more protracted and complicated visits. Finally, while all interviewees felt DAXC had significant potential, and several physicians lavished the tool with effusive praise, others did not find the current iteration of DAXC an improvement over their own established processes for clinical documentation.

Every patient encounter requires documentation, and this is often considered to be a burdensome part of a physician’s job. DAX Copilot has the potential to simplify the creation of a visit note. Using DAX Copilot may also enable the physician or APP to focus entirely on the conversation with the patient alleviating the need to take notes or document in the EHR during the encounter. This could reduce the cognitive burden for the physician and improves the patient/physician interaction during the visit.

At the time of writing, AH has negotiated a contract with the DAXC vendor to expand to 2500 licenses over the next year. We will use DAXC in multiple specialties, and will include physicians, APPs, and residents as users. Furthermore, we will endeavor to research various aspects of the impact of this tool with a focus on physician and APP wellness. One of our goals will be to identify those physicians and APPs who benefit most from this type of intervention so that we can preferentially offer them support using this tool.

The semi-structured interviews were part of a larger evaluation for our enterprise, which informed the decision of expanding utilization of the tool across our enterprise. The decision to pursue expanded use of this tool was based primarily on the subjective benefit that a majority of our users reported. This intervention was intended to support physician and APP wellness, and the reduced time spent documenting notes, and especially the reduced need to remember details were perceived positively by our intervention group, thus fulfilling our primary objective. We have found the process of onboarding physicians and APPs to the use of DAXC was very easy and intuitive. When we first introduced the tool to our study group we employed at-the-elbow support, however, with subsequent expansion we have used only in-app training and virtual support “office hours,” and this has worked well. A clear benefit of the DAXC product was scalability, demonstrated by our successful expansion of this technology across our large enterprise.

## CONCLUSION

The results of this study outline the significant potential of DAXC, as well as the current limitations of the technology. Interview data suggests that the present iterations of the tool may not yet be suitable for every type of physician encounter, but for some interviewees, DAXC has already considerably reduced the time spent on documentation.

## Supplementary Information

Below is the link to the electronic supplementary material.

Supplementary file1 (DOCX 30 KB)
