# Supplementary material for: Can the Administrative Loads of Physicians be Alleviated by AI-Facilitated Clinical Documentation?
Source: J Gen Intern Med. 2024 Jun 27;39(15):2995–3000. doi: 10.1007/s11606-024-08870-z (PMC11576703; doi:10.1007/s11606-024-08870-z)
Supplement: Supplementary file 1 — Supplementary file1 (DOCX 30 KB) [file 11606_2024_8870_MOESM1_ESM.docx]

**Interview Learning Objectives (Research Questions):**

- **Primary:** How has using DAX Express impacted physicians professional and personal lives?
- **Secondary:** What needs remain unmet by DAX Express? (for purposes executing the functional job “Document notes after patient visit”)

**Opening/Consent:**

“We only have 30 minutes, and we want to use your time wisely, so we’ll get right to it. We do have a semi-structured set of questions to cover with you. This interview is part of a qualitative evaluation of DAX Express. The interview will be recorded, but any identifying information will be redacted in the transcription process. Recordings will not be shared beyond the interview team. If there are questions you don’t wish to answer, please let me know and we will skip them. You can also stop, or terminate the interview entirely, at any point.”

Then ask if the provider has any questions for you and if it’s alright to start recording.

**Potential Provider Interview Questions:**

- Seek a basic understanding of how they performed this functional job pre-DAX Express
  - What are you seeking to accomplish when documenting notes after a patient visit?
  - When do you do this task? What does that process look like?
- So, we understand you’ve been using DAX Express for at least six weeks, maybe more. What has changed for you now, what is different about your day?
  - What do you want to do more of, or less of? Did DAX Express help you do more of ____ and less of ____?
  - Has anything changed for the care team?
  - Has anything changed between you and the patient?
- Are there any aspects of the tool that you are struggling with now?
  - Potential probing question – What issues, if any, might arise when interacting with the patient using this tool?
- How do you feel when using DAX Express? Probe to understand their language.
- If the tool performed perfectly, what would you love the outcome to be for you?
- What would cause you to stop using DAX Express?
- Is there ever an instance when you wouldn’t want to use DAX Express?
- How would you feel if you were not able to use DAX Express?
  How would this affect you?

**Key Skills on How to Talk so People will Talk (from *Deploy Empathy* by Michele Hansen)**

1. Use a gentle tone of voice and put them at ease. If you’re on camera or in person, be aware of your facial expressions and body language.
2. Validate them – but don’t agree, congratulation them or express your opinion; examples:
   1. “I can see what you’re saying/why you did that”
   2. “That makes sense”
3. Leave pauses for them to fill – don’t be afraid of a bit of silence, let them form thoughts
4. Mirror and summarize their words – don’t say “I” when you do it. Instead of “I’m hearing…”, say “It sounds like…”
   1. Interviewee: “Wow, I’ve had such a busy day”; Interviewer: “You’ve had a busy day”, or “You have a lot going on” This gives them the opportunity to elaborate.
5. Don’t interrupt
6. Use simple wording
7. Ask for clarification, even when you don’t need it
8. Don’t explain anything or get defensive (about our products or services)
9. Don’t negate them in any way – build upon what they say; the notion of “Yes, And”; avoid saying things like “Yes, but”, “Well, but”
10. Let them be the expert – don’t ever correct them
11. Use their words and pronunciation
12. **Ask about current or past behavior (this is a big one!)**
    1. People are notoriously unreliable when talking about what they might do in the future
    2. Rather than asking “what are you struggling with” or “what are your pain points”, have people walk you through a process or ask how long something takes or who they work with to get something done
13. **Be a rubber duck** – when someone is telling you about their problems or struggles, don’t solve them for them. Listen, ask questions, enable them to solve on their own. (This is a good behavior for real life!)
